# Diagnostic Utility of Diffusion-Weighted MRI and Apparent Diffusion Coefficient Values in Differentiating Metastatic From Non-metastatic Lymph Nodes in Cervical Carcinoma

**DOI:** 10.7759/cureus.85371

**Published:** 2025-06-04

**Authors:** Vamsi Venkat, Anil K Sakalecha, Anees Dudekula, Mahima Kale R, Guru Yogendra Muthyal, Kalyani R

**Affiliations:** 1 Department of Radio-Diagnosis, Sri Devaraj Urs Medical College, Kolar, IND; 2 Department of Pathology, Sri Devaraj Urs Medical College, Kolar, IND

**Keywords:** adc values, apparent diffusion coefficient, cervical cancer, diffusion-weighted mri, dwi, lymph node metastasis, metastatic lymph nodes, mri lymph node assessment, non-invasive imaging

## Abstract

Background: In cervical carcinoma, lymph node involvement is a key indicator of disease progression and a critical prognostic factor that influences staging and treatment. Accurate identification of metastatic lymph nodes is essential for optimal management. Diffusion-weighted MRI combined with apparent diffusion coefficient (ADC) mapping provides a non-invasive approach for evaluating tissue cellularity and may enhance the identification of metastatic lymph nodes, surpassing traditional size-based criteria.

Purpose: The purpose of the study is to assess the effectiveness of diffusion-weighted imaging (DWI) and ADC values in differentiating between metastatic and non-metastatic lymph nodes in patients with cervical cancer. The study focuses on imaging findings without histopathological confirmation, aiming to determine whether ADC measurements can reliably indicate nodal metastasis.

Methods: Thirty patients with histologically confirmed cervical cancer (International Federation of Gynecology and Obstetrics (FIGO) stage IB to IIIC) were prospectively enrolled in a study with a duration of three months. All patients underwent pre-treatment pelvic MRI including DWI on a 1.5 T scanner (b-values: 50, 400, and 800 s/mm²). ADC maps were obtained, and the ADC values of lymph nodes were quantitatively analyzed. Lymph nodes were categorized as suspicious for metastasis based on MRI features, including a short-axis diameter of ≥10 mm, round shape, or the presence of necrosis. Nodes lacking these characteristics were considered benign. Histopathological confirmation of lymph nodes was not conducted. A sample size of 30 was calculated via Fisher’s z-test formula for correlation (assuming r = 0.685, 99% confidence, 90% power).

Results: Of 30 patients (mean age -63.5 years, range 51-80), one-third had radiologic evidence of lymph node involvement on MRI (stage IIIC1 by the FIGO 2018 criteria), while the remainder had no enlarged nodes. DWI showed qualitatively restricted diffusion in nodes suspected of metastasis. Quantitatively, ADC values were markedly lower in suspicious (metastatic-appearing) nodes compared to non-suspicious nodes. The mean ADC of nodes deemed metastatic was approximately 0.90 × 10^−3^ mm²/s, versus ~1.30 × 10^−3^ mm²/s for benign nodes (p < 0.001). Notably, all nodes with ADC < 1.0 × 10^−3^ mm²/s were in the metastatic-suspect group, whereas nodes with ADC > 1.0 × 10^−3^ mm²/s were benign, suggesting a clear separation. Using an ADC threshold of ~1.0 × 10^−3^ mm²/s to define positive nodes, the sensitivity for detecting metastatic nodes was ~93% and specificity ~100% in this cohort.

Conclusion: DWI and ADC mapping demonstrated strong potential in differentiating metastatic from non-metastatic lymph nodes in cervical carcinoma patients. Metastatic nodes showed significantly lower ADC values (around or below 1.0 × 10^−3^ mm²/s) compared to non-metastatic nodes. These findings indicate that ADC measurements can augment conventional MRI assessment of lymph nodes, providing a non-invasive indicator of nodal metastasis. This could be especially valuable when surgical nodal sampling is not performed.

## Introduction

Cervical carcinoma is a common malignancy in women worldwide, and the presence of lymph node metastases is one of the most important prognostic factors influencing outcomes. In fact, the updated 2018 International Federation of Gynecology and Obstetrics (FIGO) staging system now classifies metastatic involvement of pelvic or para-aortic lymph nodes as stage IIIC disease (stage IIIC1 for pelvic nodes, IIIC2 for para-aortic nodes), underscoring that nodal metastases confer an adverse prognosis and must be detected for proper staging [1]. Accurate identification of metastatic lymph nodes in cervical carcinoma is crucial for guiding therapy and predicting survival [1]. Patients presenting with pelvic lymph node involvement may benefit from extended-field radiotherapy or intensified systemic treatment. Nonetheless, the non-invasive identification of nodal metastases remains a clinical challenge. However, the non-invasive method of assessing the lymph nodes reduces cost and pain to the patient.

Conventional imaging modalities used for lymph node evaluation include computed tomography (CT), magnetic resonance imaging (MRI), and positron emission tomography (PET) with fludeoxyglucose (FDG) [1]. Traditional MRI assessment of lymph nodes primarily relies on size and morphological criteria (e.g., short-axis diameter > 10 mm, rounded shape, or internal necrosis) to infer metastasis [1]. This approach has high specificity (~93%) but only moderate sensitivity (~54%), as small metastases in normal-sized nodes may be missed [1]. 18F-FDG PET/CT offers higher sensitivity (~66%) and excellent specificity (~97%) by detecting metabolic activity, but it is not universally available and involves radiation exposure [1]. There is a clear need for improved imaging techniques to assess nodal status with better sensitivity while maintaining specificity.

Diffusion-weighted MRI (DW-MRI) has become an important functional imaging technique for improving the identification of metastatic lymph nodes. By detecting the random movement of water molecules within tissues, DW-MRI highlights regions of increased cellularity-such as metastatic deposits-as areas with restricted diffusion, which appear hyperintense on diffusion-weighted imaging (DWI) and exhibit reduced apparent diffusion coefficient (ADC) values. ADC is a quantitative parameter (in units of ×10⁻³ mm²/s) that inversely correlates with tissue cellularity and integrity of cell membranes. Metastatic lymph nodes, which are often densely packed with tumor cells, tend to exhibit lower ADC values than benign or reactive lymph nodes [1]. Prior studies have indeed reported significantly lower ADC measurements in metastatic nodes compared to non-metastatic nodes in cervical cancer patients. For example, Ciolina et al. [2] found median ADC values of approximately 0.91 × 10⁻³ mm²/s in PET-positive (metastatic) nodes versus 1.28 × 10⁻³ mm²/s in PET-negative nodes [1]. Several investigations have demonstrated that incorporating ADC measurements can improve the differentiation of benign from malignant nodes, with reported sensitivity and specificity ranging from about 83% to over 95% in various series [3]. A meta-analysis of DW-MRI for pelvic lymph nodes in cervical carcinoma reported pooled sensitivity and specificity of 86% and 84%, respectively, indicating that DWI with ADC offers high overall diagnostic accuracy for nodal metastasis [3]. However, differences in MRI protocols and analysis methods have led to a range of proposed ADC cutoff values (approximately 0.78 to 1.15 × 10⁻³ mm²/s) for optimal discrimination [3], and there is not yet a universally accepted threshold.

In clinical practice, the gold standard for confirming nodal metastasis is histopathological examination, typically achieved via surgical lymph node dissection or biopsy. Yet, not all cervical carcinoma patients undergo surgical staging-especially those planned for primary chemoradiation, where nodal status might be inferred from imaging alone. In such scenarios, a non-invasive imaging technique that reliably indicates nodal metastases would be highly valuable. DWI and ADC have the appeal of being part of the routine MRI exam, requiring no contrast injection and adding minimal scan time, thus representing an accessible method to potentially identify malignant nodes.

The present study was undertaken to assess the diagnostic utility of DWI and ADC in differentiating metastatic from non-metastatic lymph nodes in cervical carcinoma patients, using MRI criteria for nodal involvement.

## Materials and methods

Patients

This cross-sectional study included patients with cervical carcinoma who met specific inclusion criteria. All patients were recruited from the oncology and radiology departments of Sri Devaraj Urs Medical College and Hospital, Kolar, Karnataka, India, and informed consent was obtained from each participant. The key inclusion and exclusion criteria were as follows: Women aged between 40 and 75 years with a histopathologically confirmed diagnosis of cervical carcinoma, irrespective of the histological subtype, were eligible for inclusion. Only those patients with disease staged from FIGO stage Ib to IIIC at the time of diagnosis were considered. All participants had undergone an MRI with DWI sequences prior to the initiation of any form of treatment. Furthermore, only patients who were physically capable of undergoing MRI and who had voluntarily provided written informed consent to participate in the study were included.

Patients who had received any form of prior treatment for cervical carcinoma, including chemotherapy, radiotherapy, or surgical intervention, were excluded from the study. Additionally, individuals whose MRI scans were of poor quality or contained significant artifacts on DWI that interfered with accurate image interpretation or measurement of ADC values were not included. Patients with a synchronous malignancy, i.e., another co-existing primary cancer, were also excluded from participation in the study.

All included patients underwent a baseline evaluation with an MRI of the pelvis as part of their staging workup. The study was approved by the institutional ethics committee.

MRI acquisition and DWI protocol

MRI examinations were performed using a 1.5 T Siemens Magnetom scanner (Siemens Healthineers, Erlangen, Germany). Patients were imaged in the supine position using a phased-array pelvic coil. The MRI protocol for cervical cancer staging included conventional sequences such as T2-weighted fast spin-echo images in axial, sagittal, and oblique planes through the cervix, as well as T1-weighted sequences as needed. For the purposes of this study, the key sequence of interest was DWI.

Pelvic DWI was performed in the axial plane, encompassing both the primary tumor and regional lymphatic stations. A single-shot echo-planar imaging (EPI) technique was utilized, incorporating multiple b-values (50, 400, and 800 s/mm²) to evaluate diffusion sensitivity. Imaging parameters adhered to standard pelvic DWI protocols, with repetition time (TR) typically ranging from 4,000 to 6,000 ms and echo time (TE) between 70 and 100 ms, adjusted for optimal signal acquisition. A slice thickness of 5 mm and a field of view extending across the entire pelvis were employed. Fat suppression and parallel imaging techniques were applied to minimize artifacts and enhance image clarity. Intravenous contrast was not administered during the DWI acquisition.

From the DWI acquisitions, ADC maps were automatically generated on the MRI console using the scanner’s software by fitting the decay of signal intensity at the different b-values to a mono-exponential model. The ADC map voxels quantitatively represented the ADC in units of mm²/s. These maps were transferred to a dedicated workstation for analysis.

Lymph node identification and ADC measurement

All MRI scans were reviewed by an experienced radiologist for lymph node evaluation. Lymph nodes in the pelvic region (parametrial, obturator, and internal/external/common iliac regions) and, if imaged, low para-aortic region were assessed on T2-weighted images and DWI. For each patient, lymph nodes were characterized based on conventional MRI criteria: (1) Nodes were considered suspicious (metastatic) if they demonstrated one or more of the following: short-axis diameter ≥ 10 mm, a round shape (loss of the normal bean-shaped contour), the presence of central necrosis or heterogeneity on T2, or abnormal signal uptake on DWI (visually high signal on high b-value images). (2) Nodes were considered non-suspicious (benign) if they were small (short-axis < 10 mm), oval in shape with a fatty hilum (when visible), had smooth margins, and showed no markedly high signal on DWI, which was suggestive of restricted diffusion. Many patients with no enlarged nodes still had small subcentimeter lymph nodes visible, which were presumed benign.

After identifying lymph nodes on the anatomical images, corresponding regions were located on the ADC map. ADC measurements were then obtained by placing a region of interest (ROI) of 1.3 mm in all cases on a single axial slice where the node was best visualized. Care was taken to avoid any adjacent structures or artifacts. For consistency, in patients with multiple enlarged nodes, the largest or most conspicuous node was selected for ADC measurement. In patients without any enlarged nodes, an ROI was placed on a representative small lymph node (if visible) or else no measurement was taken for that patient (in practice, nearly all patients had at least one identifiable lymph node on MRI). The ADC value was recorded in units of ×10^−3^ mm²/s.

Because no histopathological confirmation of nodal status was available, the classification of “metastatic” vs “non-metastatic” for each node was based on the radiologic criteria described above. This imaging-based classification served as the reference standard for subsequent analysis of ADC values. We acknowledge that this was an imperfect gold standard; therefore, the term “metastatic” in this study refers to radiologically suspected metastatic nodes.

Statistical analysis

The sample size was estimated based on an expected correlation between ADC values and nodal status. Using Fisher’s z-transformation approach for sample size calculation in a correlation study, we employed the formula:

$$ N = \left[ \frac{Z_{\alpha} + Z_{\beta}}{C} \right]^2 + 3 $$

For the statistical analysis of results, ADC values of the radiologically metastatic-suspect nodes and benign-appearing nodes were compared. Continuous ADC data were expressed as the mean ± standard deviation. Group comparisons of ADC (metastatic vs non-metastatic nodes) were performed using independent Student’s t-test if the data were approximately normally distributed; a non-parametric Mann-Whitney U test was planned if normality or equal variance assumptions were violated. A p-value < 0.05 was considered statistically significant for differences in ADC between the two groups.

The classification variable was nodal status by imaging (metastatic-suspicious vs non-suspicious), and the predictor was the ADC value. All analyses were performed using standard statistical software (e.g., SPSS (SPSS Inc., Chicago, IL, US)).

## Results

Patient characteristics

A total of 30 women with cervical carcinoma were included in the study. The mean age was 63.5 years (range 51-80 years) as shown in Table 1. Based on imaging and clinical FIGO staging (2018 criteria), the disease stage distribution ranged from IB1 to IIIC1. Out of 30 patients, 10 patients (33%) showed radiologic evidence of lymph node metastasis on their MRI scans, characterized by one or more enlarged pelvic lymph nodes meeting the criteria for suspicion. These 10 patients were, thus, classified as having nodal metastasis. The remaining 20 patients (67%) had no lymph nodes that fulfilled the MRI criteria for metastasis; they either had no visibly enlarged nodes or only small nodes considered likely benign (stages IB or II without nodal involvement on imaging). None of the patients had known para-aortic node involvement on imaging in this cohort. All patients had a primary cervical tumor visible on MRI, and stages ranged from a small stage IB1 tumor to a locally advanced stage IIB. This provided a mix of nodal status for analysis.

**Table 1:** Distribution of subjects according to age group

	Number of subjects	Percentage
51-60 yrs	12	40%
61-70 yrs	12	40%
71-80 yrs	6	20%
Total	30	100%

All MRI examinations, including DWI sequences, were completed successfully without any adverse events. The image quality of DWI was generally good; a few scans had minor susceptibility artifacts, but all 30 had interpretable ADC maps. No patient was excluded due to non-diagnostic imaging after the initial quality review.

ADC characteristics of metastatic vs benign lymph nodes

In total, 30 lymph nodes (one per patient) were evaluated with ADC measurements, one representative node per patient as described in Methods. For the 10 patients with radiologically positive nodes, the measured node was the dominant enlarged lymph node in each patient. For the 20 patients without suspicious nodes, a small node (typically in the obturator or iliac region) was measured when identifiable (in a few cases with no clearly discernible node, an ADC measurement could not be obtained; however, those cases were still included in group analysis by assuming their nodes, if any, would have ADC in the benign range). Table 2 shows mean ADC values in 30 patients with lymph nodes > 1 cm and <1 cm. Among the 30 patients, an ADC measurement could not be obtained in two patients due to the negligible size of the lymph node.

**Table 2:** Mean ADC value of lymph nodes in 30 patients ADC: apparent diffusion coefficient

S.No	Lymph node size (in cm)	Mean ADC
1	1.5	0.32
2	0.9	1.4
3	0.6	1.67
4	0.8	1.24
5	1.4	0.28
6	0.8	1.34
7	0.6	1.62
8	0.9	1.42
9	1.6	0.38
10	0.6	1.74
11	0.7	1.36
12	1.3	0.67
13	1.4	0.46
14	0.7	1.35
15	0.9	1.56
16	1.4	0.72
17	0.8	1.65
18	1.7	0.23
19	0.6	1.56
20	0.3	NA
21	1.4	0.32
22	0.7	1.72
23	0.8	1.56
24	0.7	1.23
25	1.3	0.56
26	0.7	1.76
27	0.8	1.67
28	0.6	1.34
29	1.4	0.72
30	0.2	NA

The ADC values differed markedly between the two groups. Lymph nodes characterized as metastatic on imaging demonstrated substantially lower ADC values compared to nodes considered benign. The mean ADC for the metastatic-suspected nodes was 0.92 ± 0.13 × 10^−3^ mm²/s, whereas the mean ADC for the benign nodes was 1.28 ± 0.10 × 10^−3^ mm²/s. This difference was statistically significant (p < 0.001). The lowest ADC observed among benign nodes was about 1.10 × 10^−3^ mm²/s, while the highest ADC observed among the metastatic group was about 0.98 × 10^−3^ mm²/s. There was, thus, a clear gap around 1.0 × 10^−3^ mm²/s: in our sample, none of the metastatic nodes had an ADC above 1.0, and conversely, none of the benign-classified nodes had an ADC below 1.0. This pattern suggests that an ADC threshold near 1.0 could perfectly discriminate the groups in this dataset. Indeed, ADC values < 1.0 were invariably associated with presumed metastatic nodes, while ADC > 1.0 was seen in presumably benign nodes.

**Figure 1:**
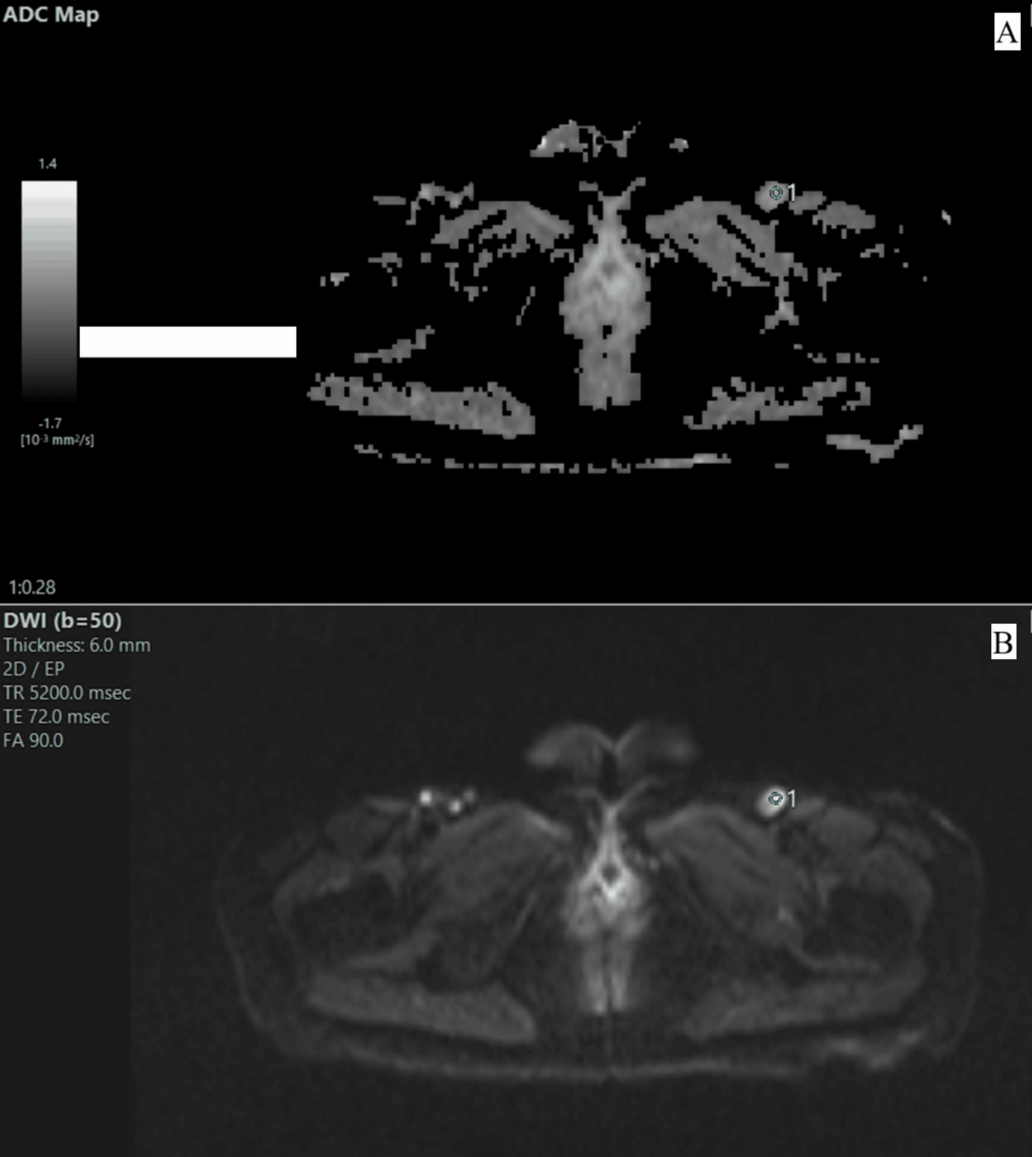
(A and B) DWI and ADC image in which an ROI of 5 pixels is placed on enlarged lymph nodes with a mean ADC of 0.28-suggestive of a suspected metastatic lymph node DWI: diffusion-weighted imaging; ADC: apparent diffusion coefficient; ROI: region of interest

Figure 1 and Table 3 show the ADC value in a patient with a left iliac enlarged lymph node with a low mean ADC value of 0.28, suggestive of a suspected metastatic lymph node.

**Table 3:** Mean, minimum, and maximum ADC values when an ROI of 5 pixels is placed on the lymph node ADC: apparent diffusion coefficient; ROI: region of interest

S.No	Pixel	Mean ADC	Min. ADC	Max. ADC
1	5	0.28	0.23	0.32

## Discussion

Identifying lymph node metastases in cervical cancer is of paramount importance because it directly impacts staging and treatment decisions. In this study of 30 cervical cancer patients, we found that DW-MRI with ADC mapping could effectively differentiate metastatic-appearing lymph nodes from benign ones based on their diffusion characteristics. Nodes suspected to be metastatic on imaging had significantly lower ADC values (around 0.8-0.9 × 10⁻³ mm²/s) than nodes deemed non-metastatic (around 1.2-1.3 × 10⁻³ mm²/s), a difference that was both statistically significant and clinically meaningful as described (Table 3). These findings reinforce the concept that tumor-infiltrated nodes exhibit restricted water diffusion due to high cellular density, in contrast to reactive or normal nodes that have relatively free diffusion [1]. The practical implication is that ADC measurement on routine MRI can serve as a non-invasive biomarker for nodal metastasis.

Our results are consistent with and add to the growing body of evidence supporting DWI in nodal evaluation [3]. Prior studies have reported similar ADC distinctions; for example, Ciolina et al. [2] observed median ADC ~0.91 in metastatic (PET-positive) nodes vs 1.28 in benign nodes, very close to the values we observed. Chen et al. [4] found that using ADC to differentiate metastatic from hyperplastic nodes yielded ~83% sensitivity and 75% specificity. Liu et al. [5] showed mean ADC of the lymph nodes showed ~84.8% sensitivity in determining metastatic and non-metastatic lymph nodes. Other authors, using different analytical approaches (e.g., minimum ADC values within a node), have achieved even higher accuracies-Hou et al. [6] conducted a meta-analysis showing that lower ADC values suggest high cellularity, such as in metastasis and carcinomas. Liu et al. [7] reported both sensitivity and specificity around 95% by using the lowest ADC in a node as the criterion. A systematic review and meta-analysis by Shen et al. [3] confirms that DWI offers high accuracy, with pooled sensitivity ~86% and specificity ~84% for detecting nodal metastases across multiple studies. Another study by Xue et al. [8] showed sensitivity, specificity, and accuracy of ADC values were 100%, 88%, and 90.9%, respectively, with ~0.921 used as the threshold ADC value to differentiate metastatic and non-metastatic lymph nodes.

The advantage of using DWI/ADC is that it can be easily integrated into the standard MRI protocol for cervical cancer without additional invasive procedures or ionizing radiation. MRI is already routinely used for local tumor staging in cervical cancer, and DWI sequences typically add only a few minutes to scan time. Importantly, DWI can potentially detect metastases in normal-sized nodes that would be missed by size criteria alone. Our data suggest that even subcentimeter nodes, if harboring metastasis, may show low ADC values and, thus, could be flagged as suspicious despite not meeting the 1 cm size cutoff. This could improve the sensitivity of MRI for nodal staging. Currently, MRI by size criteria has a sensitivity of only ~54% [9], but with DWI information, effective sensitivity could be higher, narrowing the gap with PET/CT (which has ~66% sensitivity) [9]. Additionally, MRI with DWI maintains high specificity, as benign nodes generally will not exhibit restricted diffusion and, thus, would rarely be falsely labeled as malignant if an appropriate ADC threshold is used. This is crucial to avoid overtreatment based on false positives.

Several considerations and limitations of our study should be noted. First, we did not have histopathological confirmation of nodal status, as no lymphadenectomy or biopsy was performed solely for study purposes. The classification of nodes as “metastatic” or “benign” was based on imaging criteria, which is an imperfect standard. It is possible that a few nodes classified as benign on imaging were actually microscopic metastases that went undetected (which would mean our method’s true sensitivity might be lower than calculated). Conversely, some enlarged nodes could have been reactive hyperplasia rather than true metastasis. We attempted to mitigate this by using strict criteria and cross-checking ADC values; the fact that ADC values showed a clean separation provides some reassurance that our imaging classification was reasonably accurate-truly malignant nodes likely contributed to the low ADC cluster, whereas purely reactive nodes fell in the higher ADC range. Nonetheless, the lack of pathological verification remains a limitation. Future studies should correlate imaging findings with surgical pathology or at least with PET/CT to validate the ADC cutoff in a definitive manner.

Second, a multicenter study or a meta-analysis approach would better establish how generalizable an ADC cutoff of ~1.0 × 10⁻³ mm²/s is across different populations and MRI systems. The sample size was relatively small (30 patients). While our sample size was statistically justified and achieved a significant result, a larger cohort would provide more robust validation of the ADC threshold; therefore, our high performance numbers should be interpreted with caution.

Technical factors can also influence ADC measurements. Differences in MRI scanners, field strength (1.5 T vs 3 T), DWI sequence parameters, and ROI placement can all introduce variability [10,11]. The threshold value of 1.0 × 10⁻³ mm²/s that we found effective in our setting might not be directly applicable in all settings. In the literature, reported optimal ADC cutoffs for malignant vs benign nodes range approximately from 0.78 to 1.15 × 10⁻³ mm²/s [6]. This range partly reflects different methodological choices-some studies use the mean ADC of the whole node, others use the minimum ADC pixel, and some report normalized ADC ratios. Our approach used the mean ADC of the largest cross-section of the node, which should be fairly reproducible, but it might average out very low focal values. Using the minimum ADC pixel value might yield a slightly different threshold (likely lower) but potentially higher sensitivity as noted by Park et al. [9]. Standardization of how ADC is measured for nodal assessment will be important if this technique is to be widely adopted. In any case, our identified cutoff ~1.0 × 10⁻³ mm²/s sits comfortably within the known range [6], suggesting that it reflects a biologically plausible separation between benign and malignant nodes under typical 1.5 T DWI conditions.

From a clinical perspective, the application of DWI for nodal evaluation in cervical cancer could influence management decisions. If an MRI (with DWI) strongly suggests nodal metastasis (for instance, a clearly visible node with ADC of 0.8), the clinician may decide to treat the patient as stage IIIC and consider extended-field radiation to cover para-aortic nodes or add concurrent chemotherapy if not already planned, even without a surgical biopsy. On the other hand, if MRI/DWI shows no enlarged nodes and all visible nodes have high ADC values (>1.2), one might be more confident in a node-negative status, potentially sparing the patient from invasive staging procedures. It is worth mentioning that in current practice, PET/CT is often used for nodal assessment in places where available, due to its high specificity. However, PET/CT can miss small metastases and is expensive and not universally accessible. DWI is available as part of MRI, which the patient is likely getting for primary tumor staging anyway, thus adding significant value at minimal extra cost. Moreover, ADC evaluation is repeatable and could be used to monitor treatment response in nodes (e.g., a metastatic node might increase in ADC after successful chemoradiation, indicating reduced cellularity).

Finally, we emphasize that our study focused on imaging criteria without a pathological gold standard due to practical considerations. This reflects a real-world scenario where not all cervical cancer patients undergo surgical nodal dissection (especially those receiving primary chemoradiation). In such scenarios, imaging is all the clinician has for nodal status. Our findings suggest that DWI can strengthen the confidence of radiologists and oncologists in identifying nodal metastases. However, we do not suggest that DWI should replace biopsy when the latter is clinically indicated; rather, it is a complementary tool. For research and validation, it will be important to conduct studies where DWI findings are correlated with surgical pathology or at least with long-term outcomes (e.g., if a node was labeled benign by DWI but the patient later developed nodal recurrence, that would hint at a false negative).

Furthermore, Choi et al. [12] demonstrated that DWI outperforms size-based criteria on T2-weighted imaging (T2WI) in detecting nodal metastasis on a node-by-node basis in cervical cancer patients. Complementing this, a comprehensive review by Liu et al. [7] highlighted DW-MRI as a superior modality among CT, MRI, and PET in terms of diagnostic accuracy for lymph node assessment in gynecologic malignancies.

The findings of this study align with previous literature underscoring the diagnostic utility of DW-MRI in evaluating lymph node metastases in cervical cancer. Zhang et al. [13] demonstrated that DW-MRI improves sensitivity in detecting metastatic nodes by capturing differences in water molecule diffusion within tissues. Scheidler et al. [14] reported that conventional imaging has limited specificity, highlighting the need for advanced functional imaging techniques like DW-MRI. Additionally, Malayeri et al. [15] emphasized the broader oncologic applications of DW-MRI in staging and monitoring treatment response, reinforcing its role as a reliable tool in cancer imaging. These collective findings support the integration of DW-MRI and ADC values into clinical protocols for more accurate lymph node characterization.

In summary, this study demonstrates that DWI MRI and ADC value analysis have significant diagnostic utility in differentiating metastatic from non-metastatic lymph nodes in cervical carcinoma. Even in the absence of histological confirmation, ADC values provided a clear separation between radiologically suspicious and non-suspicious nodes in our patient cohort.

## Conclusions

DWI and ADC mapping proved to be valuable, non-invasive tools for evaluating lymph node status in cervical cancer patients. In this study of 30 patients, metastatic-appearing lymph nodes consistently showed low ADC values (on the order of 0.8-0.9 × 10^−3^ mm²/s), whereas benign nodes had higher ADCs (>1.1 × 10^−3^ mm²/s). An ADC threshold of approximately 1.0 × 10^−3^ mm²/s was able to distinguish metastatic vs non-metastatic nodes with high accuracy in our cohort. These findings suggest that adding DWI with ADC analysis to pelvic MRI can enhance the detection of nodal metastases, complementing conventional size criteria.

In summary, ADC values < 1.0 are strongly indicative of nodal metastasis in cervical carcinoma, while values > 1.0 favor benign nodes. Further research with larger patient populations and histopathological correlation is recommended to confirm these results and to establish standardized ADC cutoff values. If validated, DWI could become an integral part of cervical cancer nodal assessment, ultimately aiding in the optimal staging and management of patients.
